# Methods used in prevalence studies of disrespect and abuse during facility based childbirth: lessons learned

**DOI:** 10.1186/s12978-017-0389-z

**Published:** 2017-10-11

**Authors:** David Sando, Timothy Abuya, Anteneh Asefa, Kathleen P. Banks, Lynn P. Freedman, Stephanie Kujawski, Amanda Markovitz, Charity Ndwiga, Kate Ramsey, Hannah Ratcliffe, Emmanuel O. Ugwu, Charlotte E. Warren, R. Rima Jolivet

**Affiliations:** 1 Department of Global Health and Population, Harvard T. H. Chan School of Public Health, 677 Huntington Ave, Boston, MA 02115 USA; 2 Population Council, Reproductive Health Program, 5 Rose Avenue, PO Box 17643-00500, Nairobi, Kenya; 3 School of Public and Environmental Health, Hawassa University, Hawassa, Ethiopia; 4 Department of Global Health, Boston University School of Public Health, 801 Massachusetts Ave, Crosstown Building, 3rd Floor, Boston, MA 02118 USA; 5 Averting Maternal Death and Disability Program (AMDD), Heilbrunn Department of Population and Family Health, Columbia University Mailman School of Public Health, 60 Haven Avenue, B3, New York, NY 10032 USA; 6 Department of Epidemiology, Columbia University Mailman School of Public Health, 60 Haven Avenue, B3, New York, NY 10032 USA; 7 Department of Epidemiology, Harvard T. H. Chan School of Public Health, 677 Huntington Ave, Boston, MA 02115; 677 Huntington Ave, Boston, MA 02115 USA; 8 Population Council, Ralph Bunche Rd, Nairobi City, Nairobi Kenya; 9 Management Sciences for Health, 45 Broadway #320, New York, NY 10006 USA; 10 Ariadne Labs at Brigham and Women’s Hospital and the Harvard T.H. Chan School of Public Health, 401 Park Drive, Boston, MA 02215 USA; 11 Department of Obstetrics and Gynecology, Faculty of Medical Sciences, College of Medicine, University of Nigeria Enugu Campus, Enugu, Enugu State Nigeria; 12 Population Council, 4301 Connecticut Ave NW # 280, Washington, DC, 20008 USA; 13 Maternal Health Task Force, Women & Health Initiative, Department of Global Health and Population, Harvard T.H. Chan School of Public Health, 651 Huntington Avenue, Boston, MA 02115 USA

**Keywords:** Systematic review, Prevalence, Research methods, Measurement, Disrespect and abuse, Mistreatment, Facility childbirth, Women, Pregnancy, Health

## Abstract

**Background:**

Several recent studies have attempted to measure the prevalence of disrespect and abuse (D&A) of women during childbirth in health facilities. Variations in reported prevalence may be associated with differences in study instruments and data collection methods. This systematic review and comparative analysis of methods aims to aggregate and present lessons learned from published studies that quantified the prevalence of Disrespect and Abuse (D&A) during childbirth.

**Methods:**

We conducted a systematic review of the literature in accordance with PRISMA (Preferred Reporting Items for Systematic Review and Meta-Analysis) guidelines. Five papers met criteria and were included for analysis. We developed an analytical framework depicting the basic elements of epidemiological methodology in prevalence studies and a table of common types of systematic error associated with each of them. We performed a head-to-head comparison of study methods for all five papers. Using these tools, an independent reviewer provided an analysis of the potential for systematic error in the reported prevalence estimates.

**Results:**

Sampling techniques, eligibility criteria, categories of D&A selected for study, operational definitions of D&A, summary measures of D&A, and the mode, timing, and setting of data collection all varied in the five studies included in the review. These variations present opportunities for the introduction of biases – in particular selection, courtesy, and recall bias – and challenge the ability to draw comparisons across the studies’ results.

**Conclusion:**

Our review underscores the need for caution in interpreting or comparing previously reported prevalence estimates of D&A during facility-based childbirth. The lack of standardized definitions, instruments, and study methods used to date in studies designed to quantify D&A in childbirth facilities introduced the potential for systematic error in reported prevalence estimates, and affected their generalizability and comparability. Chief among the lessons to emerge from comparing methods for measuring the prevalence of D&A is recognition of the tension between seeking prevalence measures that are reliable and generalizable, and attempting to avoid loss of validity in the context where the issue is being studied.

**Electronic supplementary material:**

The online version of this article (10.1186/s12978-017-0389-z) contains supplementary material, which is available to authorized users.

## Plain English Summary

Disrespect and abuse (D&A) of women who go to a health facility to have a baby has been identified as a widespread problem, but just how commonly it happens is not known.

In this study, a systematic review was done to find all the studies that tried to measure D&A of women during childbirth in health facilities. A direct comparison of the methods used in each paper was done, to look for sources of systematic error. The authors of these papers came together to offer lessons learned.

Over the last 5 years, several teams of researchers have tried to measure D&A of women in childbearing facilities. They used different definitions and different methods for measuring the problem. A comparison of their methods showed differences in the way that study sites and participants were chosen, as well as in the way the problem of D&A was defined and the way questions about it were asked across the five studies that were reviewed. Each of these differences may have influenced the measurement and introduced various types of bias into the results.

In conclusion, this comparative review of methods used by the first research teams to try to measure D&A points out the challenges involved. The authors recommend ways of reducing selection bias, courtesy bias and recall bias to improve future studies. Having standard definitions and using similar methods would allow comparison of prevalence measures across settings, but it is difficult to achieve because what people consider D&A is not standard in every context.

## Background

There is growing evidence of widespread disrespect and abuse (D&A) among women seeking care during childbirth in health facilities. Numerous reports document mistreatment of women during facility-based childbirth in institutions around the world, suggesting this is a phenomenon that occurs globally with differing drivers and varying degrees of severity in different contexts [1–3].

Evidence has shown that D&A can occur during any interaction between health care providers and childbearing women and is influenced by a variety of factors. These drivers include provider training and attitudes, service delivery standards, facility organization, health system leadership and governance, lack of accountability, and structural factors within societies, communities, and health systems [2, 4–6]. Research suggests that fear of mistreatment is a significant deterrent to use of health facilities for childbirth [7, 8]. A recent framework developed by the World Health Organization (WHO) to represent the essential components of quality of care for maternal and newborn health suggests that provision of care and experiences of care are equally important aspects of care quality [9]. Disrespect and abuse of women seeking maternity care is also recognized as a violation of human rights. Human rights declarations and conventions enshrine the right to freedom from harm and ill treatment [10–12] and treaty-monitoring bodies recognize maternal healthcare as a core component of states’ obligations to fulfill the right to health [13–15]. Several recent frameworks specifically highlight healthcare for women during childbirth as a human rights issue [16–18].

In 2010, Bowser and Hill introduced a framework for understanding disrespect and abuse of women during facility-based childbirth [2]. In a landscape review of reports of D&A, they proposed a classification system that grouped manifestations into seven overlapping categories: physical abuse, non-consented care, non-confidential care, non-dignified care, discrimination, abandonment of care, and detention in facilities. The authors recognized that these categories were not mutually exclusive. While the Bowser and Hill framework was developed by compiling available reports of D&A from a variety of sources and extrapolating them into categories, the resulting theoretical framework was not designed for prospective use to measure the prevalence of D&A or validated for this purpose. It was the only systematic theoretical framework available for classifying D&A until recently.

In 2014, WHO issued a statement on the prevention and elimination of D&A during facility-based childbirth calling for, among other things, further research on defining and measuring disrespect and abuse in public and private facilities worldwide [19].

To address the problem of overlapping categories of D&A, a more recent systematic review by Bohren et al. [1] updated the 2010 landscape review and proposed a revised typology. It renamed the phenomenon “mistreatment” and defined its essential dimensions slightly differently as: physical abuse, sexual abuse, verbal abuse, stigma and discrimination, failure to meet professional standards of care, poor rapport between women and providers, and health system conditions and constraints. The development of this typology was undertaken to help better inform the development and use of measurement tools and to permit evaluation of interventions [1].

Despite numerous reports including a wealth of qualitative and legal evidence documenting D&A of women during facility-based childbirth, until recently there were no available data to quantify the prevalence of these behaviors. Understanding the scope and magnitude of D&A is important for a variety of reasons. Prevalence data can provide information about the specific nature and the severity of D&A. It can build urgency, galvanizing and informing action designed to address D&A. Finally, it is essential information for designing appropriate interventions and evaluating their effectiveness to reduce or eliminate D&A.

To estimate the prevalence of D&A of childbearing women in healthcare settings located in four African countries, five cross-sectional studies were conducted between 2012 and 2014 [20–24]. Researchers in all of these studies defined D&A using the framework proposed in 2010 by Bowser & Hill [2] as a starting point to explore the magnitude of this public health problem, since their research predated the publication of the newer typology. Prevalence of D&A during childbirth had not been measured before. Thus, the first teams of researchers encountered various challenges and made different methodological decisions, which resulted in significant heterogeneity across the studies and impacted the ability to compare results. The systematic review by Bohren et al. found that inconsistent identification criteria and operational definitions, as well as differing study methods and designs, led to a degree of heterogeneity in the studies that precluded pooling the prevalence estimates via meta-analysis [2].

Variations in the reported prevalence of D&A in recent published studies may be associated with differences in study instruments and data collection methods, including the timing, setting, and modality for data collection, and variations in the constructs and operational definitions used to define each dimension of D&A. According to Bohren et al., “These variations may have contributed to the substantial differences in estimates of prevalence. The lack of standardized, comprehensive, and agreed typology, identification criteria, and operational definitions of the mistreatment of women during facility-based childbirth thus complicates further research in this important area” [1].

The aim of this study is to aggregate and present lessons learned from the first five studies that quantified the prevalence of D&A of women during childbirth. Our analysis documents and compares the decisions that were made during the design and implementation of the five prevalence studies published through August 2016 in low-income settings across four African countries. We discuss the implications of the variations in study methods for the interpretation and application of the resulting prevalence estimates. We discuss sources of potential systematic error in estimates of the prevalence of D&A and make recommendations for future research.

## Methods

### Selection of studies for systematic review

A systematic review of the literature was conducted in accordance with the PRISMA (Preferred Reporting Items for Systematic Review and Meta-Analysis) criteria [25]. PubMed and Embase were systematically searched with a restriction to English-language articles and an unrestricted publication start date through August 2016. The search strategy was designed to identify studies reporting on D&A during facility-based childbirth. The search terms were: *Disrespect and Abuse, mistreatment, childbirth, delivery, Disrespect and Abuse of childbearing women, mistreatment and women, mistreatment and childbirths, D&A and childbirths, facility delivery and D&A*. Our search string (limited to humans) included:

*(((((mistreatment[All Fields] AND (“women”[MeSH Terms] OR “women”[All Fields]) AND (“parturition”[MeSH Terms] OR “parturition”[All Fields] OR “childbirth”[All Fields])) OR “disrespect and abuse”[All Fields]) OR (dehumanized[All Fields] AND care[All Fields])) OR (humanized[All Fields] AND care[All Fields])) OR “obstetric violence”[All Fields]) OR “respectful maternity care”[All Fields] AND (((“pregnancy”[MeSH Terms] OR “pregnancy”[All Fields]) OR (“parturition”[MeSH Terms] OR “parturition”[All Fields] OR “childbirth”[All Fields])) OR maternity[All Fields]).*

**Fig. 1:**
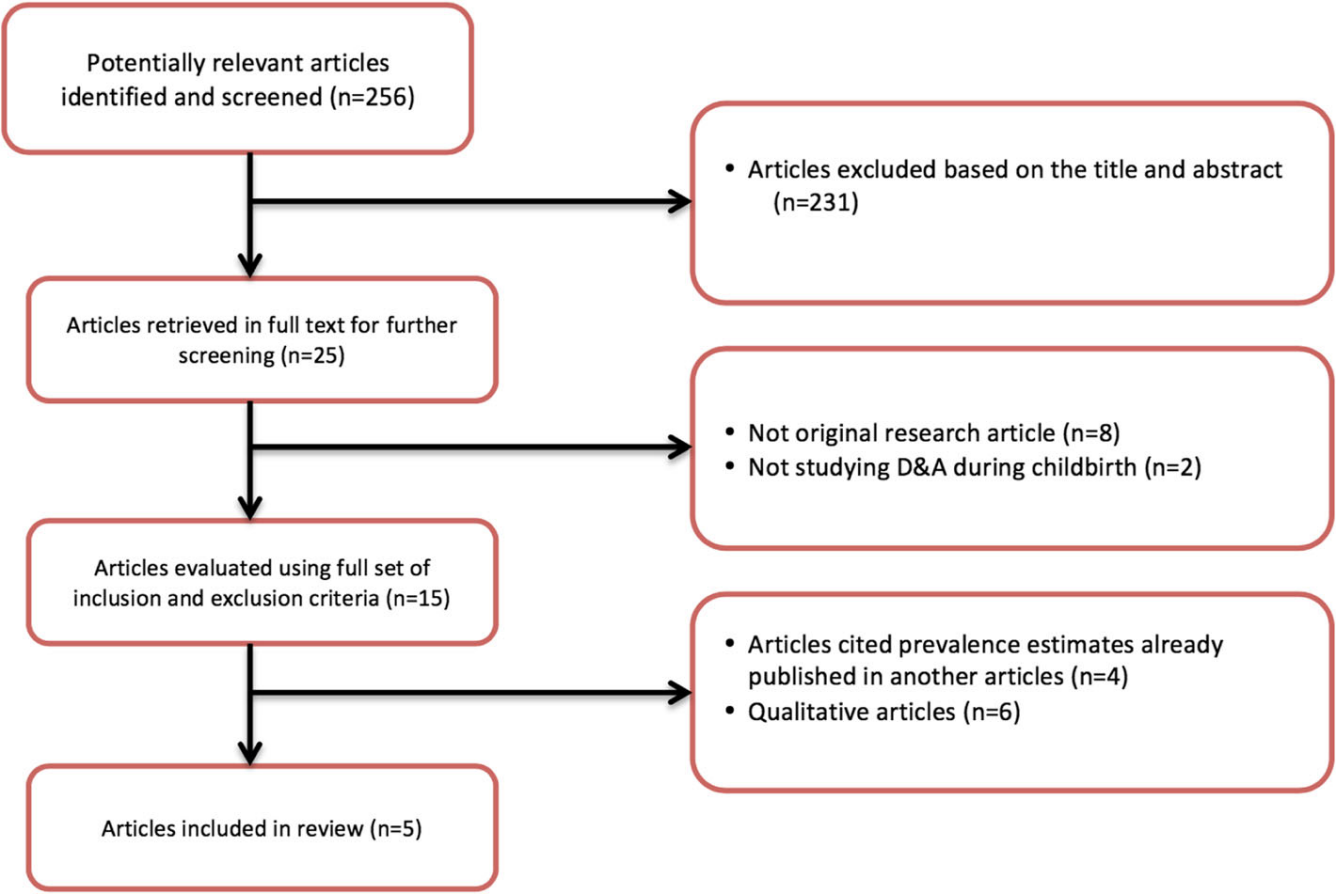
Outline of systematic review for articles on prevalence of D&A during childbirths

Eligible studies were primary research studies that focused on pregnant women and reported a prevalence measure for D&A during facility-based childbirth. Two investigators, D.S. and R.J., independently screened abstracts of all retrieved articles from PubMed and Embase and then matched full texts of all articles selected during screening against the inclusion criteria. Disagreements on eligibility were resolved by discussion. The initial search identified 256 articles on D&A. The review of titles and abstracts resulted in the exclusion of 231 papers at this stage for failure to meet inclusion criteria. The full text of the remaining 25 articles were reviewed and eight articles were excluded because they were not original research studies, while two others were eliminated because they did not report on D&A during childbirth. Of the remaining 15 articles, four were excluded because they presented prevalence measures from other published studies and another six were removed because they were qualitative studies that did not measure prevalence. Five studies fulfilled the inclusion criteria and were included in the analysis [20–24]. Figure 1 depicts a flow diagram of the search process and systematic review. Table 1 includes the characteristics of the five studies and summarizes their reported measures of prevalence.

**Table 1:** Study characteristics and summary findings

	Abuya_2015_Exploring Prevalence of Disrespect and Abuse during Childbirth in Kenya	Asefa_2015_Status of respectful and non-abusive care during facility-based childbirth in a hospital and health centers in Addis Ababa, Ethiopia	Kruk_2014_Disrespectful and abusive treatment during facility delivery in Tanzania- a facility and community survey		Okafor_2014_Disrespect and abuse in childbirth in Nigeria	Sando_2014_The Prevalence of disrespect and abuse during facility-based childbirth in urban Tanzania.	
Study Characteristics							
Study design	Cross-sectional	Cross-sectional	Cross-sectional		Cross-sectional	Cross-sectional	
Study setting	Thirteen facilities in both rural and urban Kenya	Four facilities (1 specialized hospital and 3 health centers) in Ethiopia	Eight facilities (2 district hospitals, 5 health centers and 1 dispensary) in rural, Tanzania		One referral hospital in southeastern Nigeria	One referral facility in urban, Tanzania	
Number of participants	641 women	173 women	1779 women		446 women	1914 women	
Prevalence estimates (%)	Exit Interview *n* = 641	Exit Interview *n* = 173	Exit Interview *n* = 1779	Follow-up survey *n* = 546	Written survey at 4–6 weeks postpartum *n* = 446	Exit Interview *n* = 1914	Follow-up survey *n* = 64
Overall prevalence	20	78.6	19.48	28.21	98	15	70
Physical abuse	4.2	32.9	2.9	5.08	35.7	5	52
Non dignified care	18	12.1	12.89	18.92	29.6	6	53
Non consented care	8.5	21.4	4.39	0.17	26	2	5
Non confidential care	4.3	94.8	0.06	6.16	54.5	0.2	54
Abandonment	14.3	39.3	8.53	15.54	29.1	8	52
Inappropriate demand for payment	0.9		1.78	3.07			
Detention	8.1	0.6	0.17	0.34	22	0.2	2
Discrimination		19.7			20		
Lack of privacy						2	53

### Analytical framework for comparative analysis

**Fig. 2:**
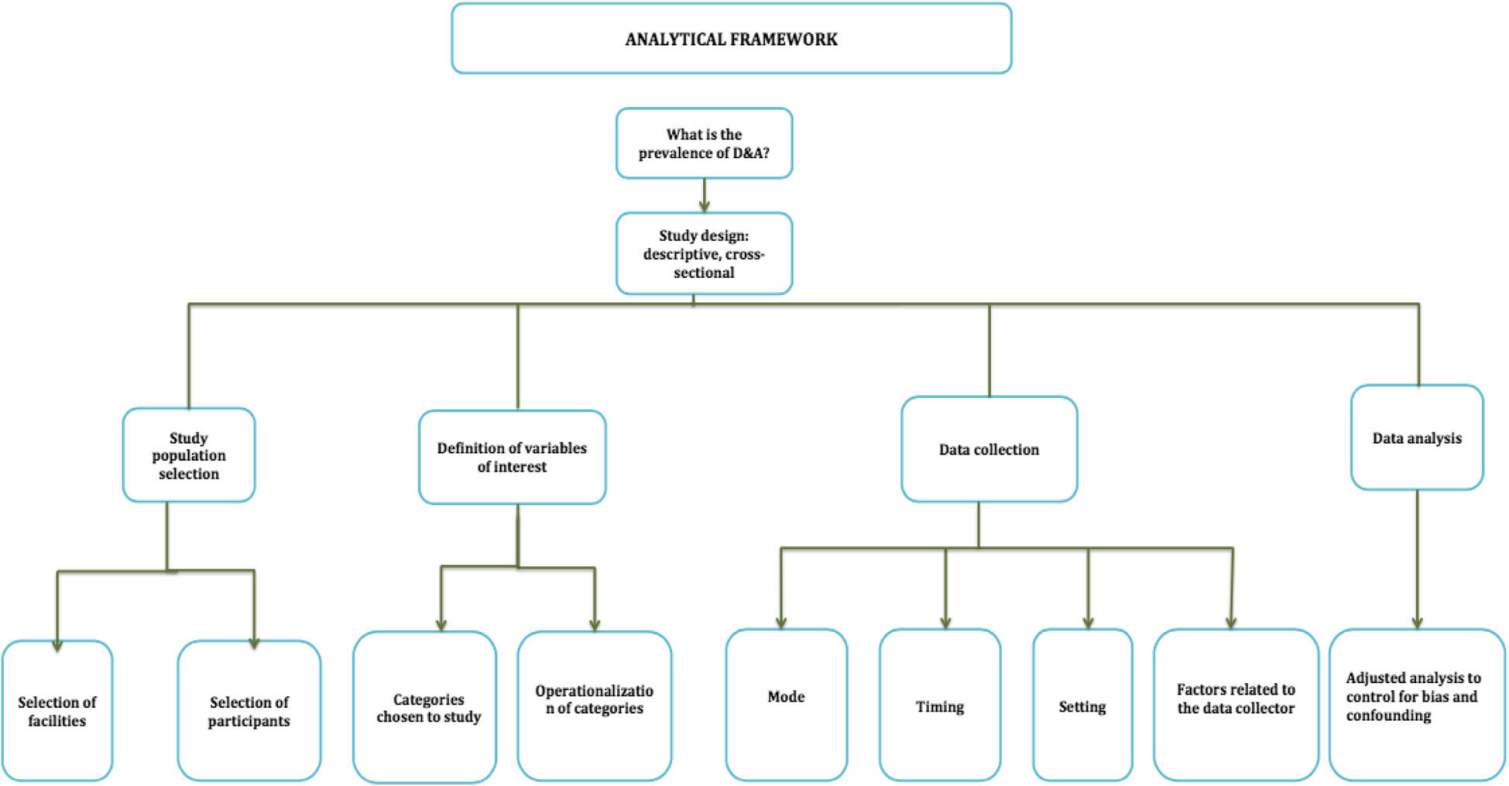
Analytical framework

Non-uniformity in study methods and designs can lead to significant heterogeneity in prevalence estimates. Therefore, we sought to systematically explore and explain possible causes of heterogeneity in the five published studies on the prevalence of D&A. We developed an analytical framework to outline all of the methodological decisions that may have been associated with systematic error, which could lead to variations in the resulting estimates of the prevalence of D&A in the studies under review (Fig. 2). This framework depicts the basic elements of epidemiological methodology in the design of prevalence studies and is composed of four main sections: study population selection, definition of the variables of interest, data collection, and data analysis. Our analysis focuses on the first three sections of the analytical framework: selection of a study population, development and operationalization of a study definition for D&A, and data collection methods. These are areas where methodological decisions may have influenced the reliability and validity of prevalence estimates, and done so in ways that are specifically relevant and potentially unique to studies of D&A. Specifically related to the study population, we examined the potential impact of sampling techniques employed in each study and characteristics of the study populations on generalizability of the findings. In defining D&A, we compared the categories of D&A used and their operational definitions in each study. Finally, we explored the impact of varying methods used during data collection such as the mode, timing, setting, and characteristics of data collectors on the validity and reliability of the prevalence estimates.

### Common types of systematic error in prevalence studies

To examine the potential for systematic error in each of the studies, we compiled a table of common types of systematic error that could result from the methodological decisions outlined in the analytical framework (Table 2). Systematic error refers to instances where the prevalence estimate differs from the true prevalence in the population to which the study is attempting to extrapolate and is synonymous with bias. The table was developed through a review of literature that included epidemiological textbooks and published methodological papers [26, 27]. The table was reviewed by the authors of the included prevalence studies, who include experienced public health professionals, statisticians and epidemiologists. The purpose was to guide an independent external review of methodological decisions made in the implementation of each study.

**Table 2:** Common types of systematic errors in prevalence studies

Type of Systematic Error	Description
Systematic Error in the Selection of Study Population	
Selection bias	This is the degree to which the survey estimate differs from the true value of the phenomenon due to the fact that study was conducted in a non-representative sample of the target population.
Response rate bias	This type of occurs when a substantial number of sampled study subjects refuse/decline to participate in the study or do not respond to a part of the study that is relevant to the outcome variable.
Measurement Errors (can be random or systematic)	
Imperfect test error	Values in the study data set do not reflect the true values of the variable of interest due to inaccurate measurement caused by poor data collection instrument It reflects validity (the degree to which the instrument measures accurately what it purports to measure).
Interviewer-related error	This is distortion of the responses given by subjects caused by behaviors of the interviewer, such as leading or cueing the subject, which influence subjects’ responses.
Courtesy/Desirability-related error	This error occurs when respondents do not report accurately on any event of interest because they don’t want to offend the person seeking their opinion.
Recall-related error	The degree to which the study value differs from the true value because of inaccurate recall of information about the variable of interest.

### Independent external review of study methods

An independent external reviewer (A.M.), who was not involved in any of the included research studies, reviewed all five studies. The independent reviewer provided an analysis of the potential for systematic error in the reported prevalence estimates in each study based on the methodological decisions made at each stage of the study design and implementation depicted in the analytical framework. She reviewed each published paper along with further details provided by the authors of each study as needed. Using the table of common types of systematic error (Table 2) as a guide, she identified potential sources of bias in each study based on methodological decisions and their implications on the prevalence estimates reported. The independent reviewer is an epidemiologist with expertise in epidemiological methods, study design, and biostatistics who has experience researching maternal health.

## Results

### Study areas and study site characteristics

The five studies included in this review were conducted in four sub-Saharan African countries. The study by Abuya et al. [20] was carried out in Kenya in 13 health facilities purposively selected from four sub-counties of Kenya: i.e. Kisumu, Kiambu, Nyandarua and Uasin Gishu. In each of these sub-counties, three facilities were selected to obtain a mix of public, private, and faith-based facilities. One additional facility was selected from Nairobi. The study by Asefa & Bekele [21] was implemented in one teaching hospital and three health centers in Addis Ababa, Ethiopia. The study by Kruk et al. [22] was conducted in Tanzania, in eight public health facilities located in two rural districts of Tanga region, including two district hospitals, five health centers, and one dispensary. The study by Okafor, Ugwu & Obi [23] was carried out in large urban referral hospital, located in Southeastern Nigeria. Finally, the study by Sando et al. [24] was also conducted in Tanzania, in one urban regional referral hospital in the Dar es Salaam region.

### Prevalence findings from each study

The overall prevalence of D&A reported across the five studies ranged from 15 to 98%. The prevalence of specific types of D&A measured also varied widely across studies. Table 1 includes the prevalence estimates reported in each of the five studies. It is important to note that while all five studies included in this review used the typology presented by Bowser & Hill [2] to develop and operationalize the study definitions of D&A, they did so in different ways. The types of indicators/questions used to measure each of the included categories of D&A varied. In addition, while all of the studies reported a measure of the overall prevalence of D&A, these summary measures were not calculated using consistent methods and did not all utilize the same categories of D&A. These differences are detailed below.

### Comparison of methods: overview

At each stage of study design and implementation depicted in our Analytical Framework (Fig. 2), the authors of the five studies included in this review made varying decisions with regard to methodology. Some of the wide variation in the ensuing prevalence estimates could be the result of systematic differences in methods or measurement error rather than a reflection of true variation in the phenomena of D&A under study.

Additional file 1: Table S1 presents the detailed results of the external review of methods by the expert reviewer, with input from the authors of the five studies in the review. The table presents a head-to-head comparison of the methods used by each team of researchers at each of the stages of study design depicted in the Analytical Framework. It includes a description of the potential impact on the validity of the ensuing prevalence measures, reflecting the effects of common types of systematic error in epidemiological studies of prevalence.

Here we summarize those results, describe the differences in methodology across the studies identified through comparative analysis, and briefly present the potential implications on the ensuing prevalence estimates.

### Selection of study facilities

Selection of facilities for inclusion in the five studies was non-random. Abuya et al. used a purposive sample of 13 facilities included in another ongoing study by the same authors [20, 28]. The facilities were selected to represent the full range of different types of facilities and levels of care in the study area, and to be similar in other respects (similar volume of births, types of providers, clientele served). The Asefa & Bekele study took place in four facilities, one specialized referral site and three health centers, with no specific selection strategy reported [21]. Kruk et al. used a purposive sample of eight facilities selected to reflect the range of delivery settings in rural Tanzanian districts [22]. The study by Okafor et al. took place in one urban referral facility, chosen because it has the highest volume of maternity care clients in the state [23]. The study by Sando et al. similarly took place in one urban referral facility, selected because of ongoing research there by the study team [24].

### Selection of study participants

There were variations in sampling techniques and exclusion criteria used for the selection of participants across studies.

Three studies reported calculating a predetermined sample size. The underlying assumptions varied. The Abuya et al. study recruited women based on sample size calculation that was performed for this research team’s larger study designed to measure the effect of a package of interventions aimed at reducing the prevalence of D&A in facilities. It was based on an assumption that 22% of women would be deterred from using a facility for childbirth due to D&A. The sample size was calculated to measure a 10% decrease of D&A, with 90% confidence with two-sided alpha of 0.005 [20]. The Asefa & Bekele study also recruited women based on a sample size calculation, based on 5% precision, 95% confidence, and a 10% non-response rate. It assumed that 13% of laboring mothers would face at least one form of disrespect and abuse during childbirth, based on a previous study conducted in three hospitals in North Ethiopia [21]. The Okafor study calculated its sample size assuming a 50% prevalence rate of disrespect and abuse during childbirth, a confidence level of 95%, a margin of error of 5%, and a nonresponse rate of 10% [23].

Most of the studies reviewed used non-random sampling to recruit study participants. Abuya et al. used convenience sampling to approach all women being discharged postpartum and recruit until the sample size was reached [20]. The Asefa & Bekele study used convenience sampling to approach women from each site in numbers proportionate to the volume of care per site to reach the targeted sample size [21]. The Kruk et al. study recruited a convenience sample of women aged 15 or older being discharged postpartum. For the community follow-up interviews performed 5–10 weeks postpartum, the researchers chose a random sample of those interviewed at exit, excluding those residing in remote areas or outside the study area [22]. Okafor et al. used a convenience sample of consecutive women presenting to a newborn immunization clinic within 6 weeks of delivery [23]. Only Sando et al. reported using random sampling, and recruited a random sample of every third pregnant women aged 18 years and older admitted to the study facility for labor and delivery services during the period of data collection [24].

Eligibility criteria differed across studies. Asefa et al. excluded women undergoing cesarean section (elective or emergency) [21]. Sando et al. excluded women who delivered by cesarean section or experienced a major complication [24]. Both of the studies that surveyed women at two different points in time (Kruk et al. and Sando et al.) excluded women residing in remote areas or outside the study area from follow up community-based interviews [22, 24]. This could also be considered (differential) loss to follow-up of women from remote geographic areas.

Non-participation rates were not consistently reported and when they were, varied widely. Abuya et al. did not report the rate of non-participation among those recruited [20]. For the Asefa & Bekele study, the non-participation rate was 9.4% [21]. Twenty-nine percent of women recruited declined participation in the study by Kruk et al. [22]. The Okafor et al. study reported a 97% response rate, thus 3% non-participation [23]. For Sando et al. the rate of non-participation was not reported [24].

### Categories of D&A chosen for study

All studies reportedly used the Bowser & Hill typology to categorize D&A for measurement [2]. These include physical abuse, non-consented care, non-confidential care, non-dignified care, discrimination, abandonment of care, and detention in facilities. However, the studies did not uniformly measure prevalence based on the original Bowser & Hill categories.

All five studies measured the prevalence of the following specific categories of D&A: physical abuse, non-dignified care, non-consented care, non-confidential care, abandonment, and detention. However, only Asefa & Bekele [21] and Okafor et al. [23] reported estimates of prevalence for discrimination, which is included by Bowser & Hill as a category of D&A [2], whereas the other three studies did not report on this category. In addition to these categories, Abuya et al. [20] and Kruk et al. [22] reported a prevalence estimate for inappropriate demands for payment, a category that they created based on construct validation through formative qualitative research, which was not included in the Bowser & Hill typology [2, 18, 19]. Also, Sando et al. [24] reported lack of privacy as a distinct dimension from non-confidentiality, while the other studies combined lack of privacy and non-confidentiality into one category of D&A, as it is described in Bowser & Hill [2, 21].

Finally, in the case of the Asefa & Bekele study [21], the Bowser & Hill categories [2, 16] were expressed in a non-uniform manner. The authors listed the rights or positive behaviors corresponding to each of the Bowser & Hill categories of D&A [17], but operationalized behaviors that constituted the violation of those rights to measure the prevalence of D&A.

### Operational definitions of categories of D&A

Across all five studies, each category of D&A was operationalized somewhat differently. The number and type of occurrences included in each category varied, as did their level of specificity.

Within categories, there were significant variations in the elements that were included in the operational definitions. As an illustrative example, in the Abuya et al. study, physical abuse was defined as being slapped, pinched, pushed, beaten or poked during childbirth [20], while in the Asefa & Bekele study, this category, which was expressed as “the woman is (not) protected from physical harm and ill treatment”, was operationalized as use of physical force, slapping, hitting, physical restraint, separation from baby without medical indication, denial of food or fluid during labor with no medical indication, not providing comfort or pain relief as necessary, or not demonstrating care in a culturally appropriate way [21]. Similar disparities in the elements included for each category are seen across all studies.

Some studies operationalized a category in general terms while others were very specific. For example, non-consented care was formulated as any treatment given without permission in the Abuya et al. study [20], while in the Sando et al. study women were asked if they received any non-consented care, including the following: tubal ligation, hysterectomy, abdominal palpation, vaginal examination, episiotomy, other [24]. A detailed comparison of the varying operational definitions used across studies appears in Table 3.

**Table 3:** Variation in the operationalization of Bowser & Hill categories of D&A across five studies

	Abuya	Asefa	Kruk	Okafor	Sando
Bowser & Hill categories					
**1. Physical abuse**	**Physical abuse**: Slapping, pinched, pushed, beaten or being poked during childbirth. Rape/sexual harassment.	**The woman is not protected from physical harm or ill treatment:** The provider used physical force/slapped me/hit me; I was: physically restrained; separated from my baby without medical indication; s denied food or fluid in labor unless medically necessitated; I did not receive comfort/pain-relief as necessary; The providers did not demonstrate caring in a culturally appropriate way	**Physical abuse:** Hitting, slapping, pushing, pinching or otherwise beating the patient; sexual harassment; rape	**Physical abuse:** Restrained or tied down during labor; Episiotomy given or sutured without anesthesia; Beaten, slapped, or pinched; Sexually abused by health worker	**Physical abuse:** Kicked, pinched, slapped, episiotomy without anesthesia, pushed, raped, other
**2. Non-consented care**	**Non-consented care:** Treatment given without permission. No permission obtained before examination for medical procedures such as tubal ligation, hysterectomy.	**The woman’s right to information, informed consent, and choice/preferences is not protected:** The provider did not: introduce himself/herself to me and my companion; encourage me to ask questions; respond to my questions with promptness, politeness, and truthfulness; explain to me what is being done and what to expect throughout labor and birth; give me periodic updates on status and progress of my labor; allow me to move about during labor; allow to assume position of choice during birth; obtain my consent or permission prior to any procedure	**Non-consented care:** Non-consent for tubal ligation; non-consent for hysterectomy; non-consent for Caesarean section	**Non-consented care:** Episiotomy; Augmentation of labor; Shaving of pubic hair; Sterilization; Cesarean delivery; Blood transfusion	**Non-consented care:** Tubal ligation, hysterectomy, abdominal palpation, vaginal examination, episiotomy, other
**3. Non-confidential care**	**Non-confidential care:** Treated in a way that violated privacy; Treated in a way that violated confidentiality. HIV status shown to others; health information discussed with non-health staff; uncovered during delivery or examination; no screens blocking view during delivery or examination; discussed her issues when other clients were listening.	**The woman’s confidentiality and privacy is not protected:** The provider did not use curtains or other visual barriers to protect me	**Non-confidential care:** Body seen by others	**Non-confidential care:** Age disclosure without consent; Provision of care without privacy; Medical history disclosure without consent; Disclosure of HIV status without consent	**Non-confidential care:** HIV status shown to others, other health information shown to others, HIV status shown to non-health staff, health information discussed with non-health staff, personal issues discussed in earshot of others, other **Lack of privacy:** Uncovered during delivery or examination, no screens blocking view during delivery or examination
**4. Non-dignified care**	**Non-dignified care:** Provider talked or used a facial expression that made you feel uncomfortable. Use of non-dignifiedlanguage such as shouting and scolding; Threats of withholding services/threatened with going to theatre, called insulting names, laughed or scorned at.	**The woman is not treated with dignity and respect:** The provider did not speak to me politely; The provider made insults, intimidation, threats, or coerced me	**Non-dignified care:** Shouting/scolding; threatening to withhold treatment; threatening comments or negative or discouraging comments	**Non-dignified care:** Blamed or intimidated during childbirth; Threatened with cesarean delivery to discourage patient from shouting; Received slanderous remarks (aspersions) from birth attendant; Scolded, shouted at, or called stupid	**Non-dignified care:** Shouted at, scolded, threatened to withhold services, laughed at or scorned, other
**5. Discrimination**	N/A	**The woman did not receive equitable care, free of discrimination:** The provider spoke to me in a language and at a language-level that I cannot understand; The provider showed disrespect to me based on any specific attribute	N/A	**Discrimination on the basis of specific patient attributes:** Denial of needed attention on the basis of ethnic origin; Denial of needed attention because of low social class; Denial of needed attention because of teen age (≤19 years); Denial of needed attention because of HIV-seropositive status	N/A
**6. Abandonment of care**	**Neglect/abandonment:** Left unattended by health workers when you needed help; Ignored when sought help for pain relief or left unattended by heath workers when they needed help.	**The woman is left without care/attention:** The provider did not: encourage me to call if needed; come quickly when I called him/her; The provider left me alone or unattended	**Neglect:** Ignoring or abandoning patient when in need; delivered alone	**Abandonment/neglect of care:** Denied companionship by the husband or close relatives; Being left unattended in second stage of labor; Birth attendant failed to intervene in a life-threatening situation; Not granted requested attention because staff was exhausted	**Abandonment:** While in labor, while delivering, while experiencing a complication, after delivery, other
**7. Detention in facilities**	**Inappropriate demand for payment**: Detention in facility for failure to pay; Request for a bribe for services	**The woman is detained or confined against her will:** I was detained in health facility against my will	**Inappropriate demand for payments:** Detention due to failure to pay; Request for bribe	**Detention in the health facility:** Discharge postponed until her hospital bills are paid; Detained in the hospital until infant’s bills are paid	**Detention:** Any

### Summary measures of overall D&A prevalence

Summary measures of D&A were not derived in the same way across all the studies. Four of the five studies reported a summary prevalence measure that was computed by tallying the number of women who reported answering “yes” to experiencing at least one of the dimensions of D&A studied (dimensions which differed across studies as noted above); in contrast, the Abuya et al. study reported a summary measure of prevalence based on self-report by asking women in one “yes/no” question if at any point during labor and delivery they were treated in a way that made them feel humiliated or disrespected [20].

### Mode, timing, and setting of data collection

The five studies collected prevalence data using a combination of different modalities, each of which could have introduced the potential for different types of systematic error into the ensuing prevalence measures.

Abuya et al. [20], Asefa & Bekele [21], and Kruk et al. [22] conducted exit interviews at postpartum discharge from the maternity unit to collect data; the study by Sando et al. [24] conducted interviews with women in the postnatal unit prior to discharge from the facility. In the studies by Kruk et al. [22] and Sando et al. [24], additional data collection took place via follow-up community interviews for a subset of the study sample at a later time. Sando et al. [24] collected follow-up data 4 to 6 weeks after delivery, while Kruk et al. [22] did so 6 to 10 weeks postpartum. In the study by Okafor et al. [23], self-administered questionnaires were given to women who presented to a childhood immunization clinic within 6 weeks of giving birth; assistance was provided for women who were illiterate). In addition to data collection via survey or interview, direct observation during labor and delivery was used by Abuya et al. [20], Kruk et al. [22], and Sando et al. [24] to collect data. However, none of them reported prevalence rates based on these data in their published studies.

The timing of data collection also varied across the studies. Timing of the collection of self-reported data introduces the potential for recall-related bias. Four studies (Abuya et al., Asefa & Bekele, Kruk et al., and Sando et al.) [20–24] conducted interviews with women shortly after giving birth. Most of these were carried out 3 to 6 hours after delivery; Abuya et al. [20] conducted interviews within 24 h of birth depending on the time of discharge. In contrast, Okafor et al. [23] conducted data collection 4 to 6 weeks post-delivery among women who presented to a clinic for immunization services for their infants.

The settings in which data were collected may also have had a systematic effect on the prevalence estimates reported in the five studies. Notably, courtesy bias is a possible source of systematic error for patient-reported data collected at the point of service. Asefa & Bekele [21] and Sando et al. [24] conducted interviews in private rooms within the maternity ward. In Abuya et al. [20], women were interviewed in the facility, in a secluded place outside the maternity ward. Kruk et al. [22] conducted interviews in a designated space outside the facility, but on the hospital grounds. Okafor et al. [23] distributed surveys in a childhood immunization clinic within the same hospital facility where women had delivered within the past 6 weeks. The women were interviewed privately in separate rooms.

Additional file 1: Table S1 presents greater detail on the types of systematic error that may have been introduced at each stage of study implementation for each of the five studies and suggests the directionality of the effect on the prevalence estimates whenever possible.

## Discussion

This paper brings together the researchers from five studies published before August 2016 that reported an estimate of the prevalence of D&A. It presents collective lessons learned on the impact of various methodological designs on the accuracy and usability of the ensuing prevalence estimates. The five published papers report a remarkably wide range in the estimated prevalence of D&A (15–98%). Given that all five studies were conducted in resource-limited settings with relatively similar maternity health care service delivery systems, this difference likely cannot be explained by differences in the study settings and study populations alone. Some degree of the observed variation could be explained by differences in the study designs, implementation processes, and operationalization of the construct of D&A. To the extent that these variations can be described and their impact on the outcomes explained, lessons can be extracted for future research to improve the reliability and validity of measures of the prevalence of D&A in facility-based childbirth. Since these first attempts to measure the prevalence of D&A, much interest in the topic of measurement in this area has arisen and new contributory work has emerged [29–31]. This paper is intended to contribute to the ongoing research in this area to further refine and standardize the measurement of mistreatment of women during childbirth in different settings.

This paper has some limitations. The systematic review of the literature did not include individual categories of disrespect and abuse from the Bowser & Hill framework (e.g. “non-consented care”) in the search terms used in the systematic review to identify articles quantifying disrespect and abuse (D&A) of women during facility childbirth. The search might have been more comprehensive if we had searched separately for specific manifestations of D&A from the chosen definitional framework.

All studies identified for comparative review of methods were conducted in Africa. In addition to the potential threat to generalizability introduced by study methods, the relative homogeneity of the study settings may limit the generalizability of their findings to countries in other geographic regions or other resource categories.

### Lessons learned

At each stage of study design and implementation, researchers make choices that have the potential to affect the outcomes of their research. Under ideal conditions, methods are chosen in order to avoid or minimize bias. In real life, this is not always possible. Furthermore, conditions sometimes arise that require researchers to weigh trade-offs in the study design based on concerns that range from the mundane, such as resource constraints, to the profound, such as ethical considerations with regard to patients’ experiences. Here we discuss the lessons learned by researchers who were the first to attempt to quantify this complex phenomenon in a variety of settings and research contexts. We conclude with recommendations for future research.

#### Selection of study facilities

Selection of study facilities and study participants has a direct impact on the validity of prevalence estimates and their generalizability. There were notable disparities in the ways each study team chose their study facilities and participants, which we have described. These differences primarily pose a threat to the applicability of these findings to other settings and to women other than those in each of these study populations, rather than on the accuracy of the estimates. If the risk of D&A in the selected facilities was different from the risk of D&A that would have been found in a sample of randomly selected facilities, conclusions about the prevalence of D&A in the study sites might not be useful for predicting risk of D&A in other facilities.

Gaining authorization to conduct research focused on abuses in the course of patient care, especially since these were among the first studies exploring D&A, was sensitive and required the researchers to establish relationships and build trust; it would have been even more difficult to gain authorization to randomly select study sites. In addition, the primary aim or research question in each study may have influenced the choices made related to the design and implementation of the study to a significant extent. For example, in three studies, i.e. Abuya et al. [20], Kruk et al. [22], and Sando et al. [32], prevalence was measured solely in order to establish a baseline for assessing the effectiveness of intervention trials prospectively. For this reason, priority was not given to choosing methods designed to ensure the generalizability of these measures beyond the implementation sites. However, to address generalizability within the region, Abuya et al. [20] chose a representative sample of facilities by level of care and geographic location across five counties. For the remaining two studies, i.e. Asefa & Bekele [21]and Okafor et al. [23], the primary aim of the study was to measure prevalence. Regardless of intent, the lack of random selection of study sites across all five studies hampers the generalizability of the findings beyond the study settings.

#### Selection of study participants

Differences were noted across studies in key components of study participant selection: sample size calculation, eligibility criteria, method of participant recruitment, reporting non-participation, and managing participant follow-up. Sample size estimation for prevalence studies is a function of expected prevalence and precision sought at a given level of confidence, where the goal is to sample a sufficient number of people to detect the population prevalence of the condition with confidence that the findings do not reflect sampling bias. The lack of previous data to inform expected prevalence in the case of D&A led to widely varying baseline assumptions that may have impacted the precision of the prevalence estimates.

Lack of randomization in the recruitment of study participants introduces potential bias into the resulting prevalence estimates. Moreover, differences in eligibility criteria may have impacted the accuracy of the prevalence estimates if women excluded from the studies based on specific characteristics or conditions were at a differential risk for D&A based on those characteristics or conditions.

Some studies excluded subjects for various reasons, including logistical reasons (for example, excluding follow up interviews with women residing outside the district or in remote areas), and ethical reasons (for example, excluding women who experienced a complication or underwent cesarean section, for whom an interview at 3–6 h postpartum would represent an undue hardship). However justifiable, such exclusions may have biased the prevalence estimates.

Exclusion of subjects who underwent cesarean section could have led to systematic variation in estimates of the prevalence of D&A in those studies if those undergoing surgical delivery are significantly more or less likely to experience D&A compared to those who have a normal course of labor and birth. For example, if greater exposure to facility care due to prolonged stay on the labor unit, referral from lower level facilities, or treatment by different personnel within the facility increases a woman’s risk of experiencing D&A, this may have affected the prevalence reported in studies that excluded women with operative delivery. This makes those rates incomparable to those reported in studies that did not make such exclusions. Because such exclusions were not a standard procedure across all five studies, it complicates the interpretation and comparability of the estimates.

Conversely, including multiparous women in studies estimating the prevalence of D&A could lead to biased estimates if previous exposure to D&A during childbirth at the same facility (or any facility) systematically increases the likelihood of normalizing the experience of D&A. It is certainly plausible that multiparous women might be less likely to perceive or report D&A during their second birth experience, if their experiences were normalized or no redress was available to them when they first experienced D&A. On the other hand, women who are more experienced (and thus may have fewer questions, or progress more quickly through labor) may experience less D&A. Whether parity affects the risk of D&A, and if so, the directionality of the effect, remains unknown. Depending on the proportion of multiparous women in the sample, estimates could be over or under estimated. Kruk et al. [22] conducted multivariable logistic regression to look at the significance of different covariates, including parity, length of stay, etc. on D&A.

Other eligibility criterion could have resulted in systematic error if the women who were excluded based on specific characteristics were at differential risk for D&A. For example, in Sando et al. [24], in order to participate in the community survey women who were recruited during their survey conducted shortly after birth were asked for a mobile phone number on which the researchers could contact them for directions to their home and to confirm consent. If women without access to mobile phones were different from those with phones in a way that affects risk for D&A, this could have biased the resulting prevalence estimates. In Kruk et al. [22] and Sando et al. [24], some women were not chosen for follow up survey due to logistical factors, such as residence in remote areas or areas outside of the catchment area, making them difficult to reach. This loss to follow-up between exit and community survey could introduce systematic error into the estimates.

Finally, if women who declined participation were systematically different from those who consented to participate, this may have impacted the reported prevalence.

#### Categories and operational definitions of D&A

Our analysis revealed the presence of various types of imperfect test-related measurement error. First, few studies reported on efforts to validate their measures. Second, even though all studies drew from the Bowser & Hill typology [2] to define the categories of D&A, there were differences across studies in the categories they chose to measure and in their operational definitions. As described, some study teams chose to collapse, disaggregate, or remove certain categories of D&A from the Bowser & Hill framework for various reasons. Categorization of D&A is to some degree subjective, and the Bowser & Hill typology is just one framework for classifying the types of D&A that have been observed [2]. This seminal framework was the only one available at the time that these five studies undertook measurement of prevalence. Each team of researchers did its best to adapt the categories for relevance and usability in context, in some cases, i.e. Abuya et al. [20], Kruk et al. [22], based on their own formative definitional work [5] and qualitative research with stakeholders to validate the constructs.

In some cases, decisions were made a priori to alter the Bowser & Hill categories in a study [2]. For example, Sando et al. [24] measured lack of privacy and non-confidential care as separate categories. As described, Abuya et al. and Kruk et al. [20, 22] created a new category, inappropriate demand for payment, which included detention in facilities for failure to pay and requests for bribes, and eliminated the category of discrimination based on qualitative research conducted in the formative stage of their study to validate the constructs defined in their study instruments.

In other cases, study teams reported that methodological issues arose during data collection and the decision was therefore made to eliminate a category based on concerns about the reliability of the data collected.

Differences in the selection and definition of categories used for quantification of D&A may reflect varying understandings across settings of what constitutes such abuse. These differences in turn affect the comparability of the summary measures of overall D&A experienced across the studies, and may lead to underestimation of total D&A if the prevalence of excluded categories of D&A was high.

Even for studies measuring the same category of D&A, the behaviors or occurrences constituting the operational definitions for those categories of D&A differed substantially. The level of specificity in the way categories of D&A were operationalized also varied substantially across studies, with some instruments inquiring about a list of specific items constituting manifestations of D&A in each category while others asked more open-ended questions about the category in general. This resulted in substantial variation in the instruments used to measure D&A across studies, differences not only in the categories measured but also in the number and range of manifestations of each category. This is not surprising, given that these were the first five studies to measure D&A and there was no guidance from previous evidence or validated instruments available for use.

Variations in the specific occurrences or behaviors that researchers chose as examples of D&A under each category resulted in researchers asking women about different phenomena (or looking for different phenomena during direct observations). This affects the ensuing prevalence measures and makes them difficult to compare, because, in effect, each study measured the prevalence of slightly different things. Additionally, the prevalence of categories of D&A in those studies for which no estimate was reported remains unknown.

Finally, aggregated summary measures of D&A were calculated in a non-standard manner across studies, making those estimates incomparable. These differences make the overall prevalence estimates in each study incomparable: in addition to the fact that Abuya et al. used a different method than the other studies to capture overall prevalence, the summary measures are incomparable because the components of D&A are very different in the four studies that used a tally.

#### Mode, timing and setting for data collection

Issues of courtesy bias, recall bias, and normalization, all resulting in potential underreporting of D&A are some of the types of systematic error related to the methods chosen for data collection that may have impacted the reported prevalence estimates in these studies.

This comparative analysis raises some interesting issues and potentially unique findings with respect to recall in the specific context of childbirth based on the mode and timing of data collection. Two of the included studies, Kruk et al. [22] and Sando et al. [24], interviewed the same women at two separate time points: shortly after delivery in or near the facility and between 4 and 8 weeks later in their homes. They found a substantial difference in prevalence estimates based on timing and setting, with higher estimates of prevalence captured on the community surveys compared with the exit surveys (Kruk et al.: 28.2% vs. 19.5%; Sando et al.: 70% vs. 15%). While recall is typically thought to decrease in accuracy over time, there are factors specific to childbirth and perhaps to the phenomenon of D&A that could challenge this assumption in this context.

There are two factors specifically related to labor and birth that could explain the differential reporting of D&A over time. First, for interviews took place within hours after giving birth, postpartum women’s recall may have been affected by extreme fatigue, high levels of adrenaline or oxytocin, low blood glucose, and competing priorities such as the desire to go home and to bring the baby to meet its father and family who could not accompany her in labor. This could lead to under-reporting of D&A. Second, while memory typically fades over time, in the specific context of childbirth women tend to go back over and review their experiences during labor and birth as a way to process what happened, telling their birth story again and again to friends and family members [33, 34]. This is a noted way of coping with intense or traumatic experiences and is also common for women in the aftermath of birth [35, 36]. Experiences of D&A could add to the naturally intense and for some women traumatic experiences related to giving birth. This, in addition to the factors described above related to the timing of data collection immediately after birth, is a factor specific to the context of childbirth that could explain the higher prevalence of D&A recorded through women’s reports collected in the community 4–10 weeks after delivery.

Moreover, the setting for data collection may be influential. It is reasonable to hypothesize that courtesy bias, including fear of repercussions if the participants believed that the researchers were affiliated with the facility and their responses might not be kept confidential, could have affected the women’s willingness to report D&A while in or near the facility in which care was provided. These factors could help explain why prevalence rates captured via surveys in or close to the health facility were lower than those rates reported through data collected via interviews with women at a later time in their own community setting. In the studies where there is no community prevalence data for comparison, the impact of data collection in the facility setting is unknown.

Traditionally, while more expensive and labor-intensive, direct observation is regarded as the gold standard for measuring observable phenomena in prevalence studies [37], because it is considered more objective than self-reported measures; however only one study, Sando et al. [24], reported on any findings based on direct observation. There are lessons related to the implementation of direct observation for measuring the prevalence of D&A that can be applied to future research.

For example, Abuya et al. [20], Kruk et al. [22], and Sando et al. [24] collected data via observation, but ultimately none reported a measure of prevalence based on these data. In the context of this comparative analysis of methods, Abuya et al. [20] and Sando et al. [24] reported that it was not possible to provide a prevalence estimate from direct observation that was comparable to the prevalence derived via exit or community surveys. This was because the tool used to collect data during observation did not match the instrument used in the exit interview questionnaire.

There are some forms of systematic error that may be associated with observation. The Hawthorne effect, in which behavior under study changes because the actors know they are being observed, is well-documented [38, 39]. Given the nature of D&A, this effect would be plausible if the care providers knew what the researchers were measuring. Observer bias could also introduce variation in the reported prevalence if inter rater reliability was not tested and some observers classified behaviors as D&A differently from others. However, the use of observation could help to offset the effect of normalization on self-reported measures of D&A, which is an intrinsic risk for all self-reported measures of prevalence across the five studies.

### Recommendations and implications for future research

In their call to action, Jewkes and Penn-Kekana rightly point out that measuring the prevalence of mistreatment of women during childbirth can be a powerful tool to help end the abuse, but that doing so well is complicated and therefore how such research is done is important [40]. The most appropriate methodology for any study depends on its aims; measures of prevalence are collected for varying purposes including contributing to the understanding of the overall scope and magnitude of D&A and evaluating the effects of interventions to address it in specific facilities. Thus, different methods may reflect different aims; and the downsides of decisions about specific methodologies must be considered in light of those primary study aims. As Freedman et al. [5] pointed out in their commentary on defining disrespect and abuse in childbirth, “To be useful in practice, the definition of disrespect and abuse requires both normative standards and experiential building blocks”. That is, depending on the intended use, a measure of the prevalence of D&A may seek to capture behaviors that all agree constitute D&A, behaviors that providers do not consider D&A but women do, and behaviors that women have normalized but others consider D&A. The optimal tools and methods for measuring prevalence of each “experiential building block” of the full phenomenon will vary.

Cost and human resource constraints affect the ability of researchers to implement the gold standard in every instance. Nevertheless, for studies that will report an estimate of the prevalence of D&A, even as a secondary aim, the following recommendations are intended to promote optimality in the design and implementation of the research to produce robust results that are reliable, valid, and comparable.

1. Lack of randomization in the selection of study sites and participants jeopardizes the application of the findings to facilities and women outside these settings. Ideally, site and participant selection in future studies should be based on methods designed to ensure no systematic differences in the study sample compared to the target population.
2. Lack of standard inclusion criteria for study participants in prevalence studies affects the comparability of resulting prevalence estimates. To estimate the population rate of D&A, all women at risk (i.e., all women receiving maternity care in the study facility) should ideally be included regardless of pregnancy outcomes. Stratified analysis could allow comparison of rates of prevalence for women with specific characteristics that may place them at differential risk for D&A, e.g., women who experienced complications or operative delivery. Stratified analysis could also help to determine whether parity systematically impacts women’s perceptions of D&A. Multivariable logistic regression can be used to assess the impact of multiple covariates including parity, length of stay, etc. on the risk of D&A.
3. Lack of standardization to ensure measurement of the same categories of D&A using the same operational definitions means that the measures of D&A are not comparable. Reliability and external validity are important concerns for researchers attempting to understand the phenomenon of D&A in general and to compare across settings. Standardization of measurement would ensure comparability of reported estimates. However, ensuring valid localized measures that capture the constructs of D&A as perceived and experienced within a specific context is also key to accurate measurement. It is therefore important to acknowledge the tension between standardization and localization in developing instruments to measure the prevalence of D&A. Use of standard categories is important for comparability, while some leeway may be needed for context-specific operationalization of those categories. The primary aims of the research may help to guide these decisions.
4. Interviewing women inside or in close proximity to the health facility where they may have experienced D&A introduces a significant risk of courtesy bias; whenever possible, conducting interviews to capture women’s self-reported experiences of D&A in another safe, neutral setting is recommended.
5. In the specific context of childbirth, recall may be poorer immediately following delivery when women are physically exhausted and have not had time to mentally process the events that occurred during labor and birth. In contrast to the typical understanding of recall deteriorating over time, in this context, women’s self-reports of D&A may be more accurate when solicited after they have had some time to process their experiences, and in a setting that is removed from the facility where they received maternity care. Future research is needed to explore this question.
6. Traditionally, direct observation is regarded as the gold standard for measuring observable phenomena in prevalence studies [41], because it is considered more objective than self-reported measures. If researchers aim to objectively measure the true prevalence of the behaviors that all would agree constitute D&A, observation by independent observers likely provides a more unbiased and accurate result. However, when the outcome of interest is women’s experiences of care, their own self-reports -- ideally using patient-developed or patient-validated measures and participatory research techniques -- are the better approach to data collection. Capturing estimates of prevalence using both approaches, with careful attention to ensure comparable instruments, offers the possibility of understanding the gap between objective and subjective constructs of D&A, i.e. the scope of normalization. Since the presence of outside observers at a woman’s birth may affect her experience, researchers studying experiences of care must ensure informed consent and respect women’s preferences and right to withdraw at any time.

Chief among the lessons to emerge from comparing methods for measuring the prevalence of D&A is recognition of the tension between seeking prevalence measures that are reliable and generalizable, and attempting to avoid the loss of validity in the context where the issue is being studied. This dilemma is germane to future research and policy, as there is a great deal of current discussion on how best to measure D&A and a search is underway for a few reliable and generalizable tracer indicators that can be incorporated into global quality of care frameworks, for example, and used for global monitoring. Our experiences highlight how complicated this task is and give real-life examples to illustrate the challenges inherent in balancing standardization versus localization in developing the “best” measures of D&A.

The search for the “true value” of the prevalence D&A requires grappling with many complex issues in addition to those of study design and methodology. These include the normalization of mistreatment of women in societies, structural inequalities and power differentials within the culture of medicine and the broader culture in which the health system resides, and health system constraints that may impact perceptions of what constitutes acceptable service and treatment of patients. The role of gender inequality as a driver of D&A is a factor that affects both recipients and providers of care. Research is needed to explore these determinants of D&A and their impact on the ability to understand the scope and drivers of the problem, as well as to intervene effectively to eliminate it. More evidence is also needed to address the lack of consensus on what constitutes the positive framing or positive construct of “Respectful Maternity Care” (RMC) and its essential components, how this construct may vary from a clinical quality of care perspective or a human rights perspective, how it may vary by context, and how best to define and operationalize it for measurement. This study aims primarily to shed light on the methodological challenges associated with quantifying D&A and to offer lessons learned to benefit future research is this area.

## Conclusions

This is the only study to date that has analyzed the methodological approaches employed to estimate prevalence of D&A in the published literature and explored the associated implications of differences in methods on the validity and generalizability of the estimates. The study underscores the need for caution in interpreting or comparing previously reported prevalence estimates of D&A during facility-based childbirth. By presenting collective lessons learned about the impact of varying methodological designs on the accuracy and usability of the ensuing prevalence estimates, this study sets the stage for more robust studies yielding prevalence estimates with high validity and generalizability. It is hoped that future researchers will find practical guidance for developing sound methodological designs to measure the prevalence of D&A that minimize the risk of systematic errors in measurement.

## Additional file

Additional file 1: Table S1. Comparison of methods used in each study and implications on prevalence estimates. (DOC 91 kb)

## Abbreviations

D&A: Disrespect and abuse
PRISMA: Preferred Reporting Items for Systematic Review and Meta-Analysis
WHO: World Health Organization
