# Supplementary material for: Methods used in prevalence studies of disrespect and abuse during facility based childbirth: lessons learned
Source: Reprod Health. 2017 Oct 11;14:127. doi: 10.1186/s12978-017-0389-z (PMC5637332; doi:10.1186/s12978-017-0389-z)
Supplement: Additional file 1: Table S1. — Comparison of methods used in each study and implications on prevalence estimates. (DOC 91 kb) [file 12978_2017_389_MOESM1_ESM.doc]

***Table S1 Comparison of methods used in each study and implications on prevalence estimates***

|  | ***Abuya.T_2015_Exploring Prevalence of Disrespect and Abuse during Childbirth in Kenya*** | | ***Asefa_2015_Status of respectful and non-abusive care during facility-based childbirth in a hospital and health centers in Addis Ababa, Ethiopia*** | ***Kruk_2014_Disrespectful and abusive treatment during facility delivery in Tanzania- a facility and community survey*** | ***Okafor_2014_Disrespect and abuse in childbirth in Nigeria*** | ***Sando_2014_The Prevalence of disrespect and abuse during facility-based childbirth in urban Tanzania*** |
| --- | --- | --- | --- | --- | --- | --- |
| 1. **Study Population** |  | |  |  |  |  |
| - 1. Selection of facilities | Non-random sampling  Purposive, representative sample | | No selection strategy reported | Non-random sampling  Purposive, representative sample | No selection strategy reported | No selection strategy reported |
| Type of related bias | Selection bias | | Selection bias | Selection bias | Selection bias | Selection bias |
| Potential impact on prevalence estimates | - Affects generalizability of findings if risk of D&A in study facilities differed systematically from general population - Some studies attempted to select a representative sample of the types of facilities in the study area but non-random sampling may have resulted in a selection of facilities in which the risk of D&A was systematically different from the population risk | | | | | |
| - 1. Selection of study participants | Non-random, convenience sampling based on sample size calculation | | Non-random, convenience sampling based on sample size calculation, sample size per facility proportionate to the volume of patients served there | Non-random, convenience sampling for exit surveys; random sample of those women selected for community follow-up survey | Non-random, convenience sampling | Random recruitment of every third pregnant woman presenting to the labor ward for admission |
| Type of related bias | Selection bias | | Selection bias | Selection bias | Selection bias | None |
| Potential impact on prevalence estimate | - Introduces risk of biased estimates due to potential systematic differences between characteristics of participants | | | | |  |
| - 1. Exclusion criteria | None reported | | Excluded women undergoing cesarean delivery (elective or emergency) | Excluded women residing in remote areas or outside the study area from follow up community-based interviews | None reported | Excluded women who delivered by cesarean section or experienced a major complication; Excluded women residing in remote areas or outside the study area from follow up community-based interviews |
| Type of related bias | None | | Selection bias | Selection bias | None | Selection bias |
| Potential impact on prevalence estimate | - Introduces risk of biased estimates if women excluded from the sample were at differential risk for D&A | | | | | |
| - 1. Non-participation rate | Not reported | | Reported 9.4% | Reported 29% | Reported 3% | Not reported |
| Type of related bias | Response rate bias | | Response rate bias | Response rate bias | Response rate bias | Response rate bias |
| Potential impact on prevalence estimate | - Non-participation may have led to over- or under-estimation depending on the reasons for declining | | | | | |
| 1. **Definition of D&A** |  |  | |  |  |  |
| - 1. Categories of D&A   *Bowser & Hill (reference)* |  |  | |  |  |  |
| - - 1. Physical abuse | 1. Physical abuse | 1. The woman is not protected from physical harm or ill treatment | | 1. Physical abuse | 1. Physical abuse | 1. Physical abuse |
| - - 1. Non-consented care | 1. Non-consented care | 1. The woman’s right to information, informed consent, and choice/preferences is not protected | | 1. Non-consented care | 1. Non-consented care | 1. Non-consented care |
| - - 1. Non-confidential care | 1. Non-confidential care | 1. The woman’s confidentiality and privacy is not protected | | 1. Non-confidential care | 1. Non-confidential care | 1. Non-confidential care |
| - - 1. Non-dignified care | 1. Non-dignified care | 1. The woman is not treated with dignity and respect | | 1. Non-dignified care | 1. Non-dignified care | 1. Non-dignified care |
| - - 1. Discrimination |  | 1. The woman did not receive equitable care, free of discrimination | |  | 1. Discrimination on the basis of specific patient attributes |  |
| - - 1. Abandonment of care | 1. Neglect/abandonment | 1. The woman is left without care/attention | | 1. Neglect | 1. Abandonment/neglect of care | 1. Abandonment |
| - - 1. Detention in facilities |  | 1. The woman is detained or confined against her will | |  | 1. Detention in the health facility | 1. Detention |
|  | 1. Inappropriate demands for payment |  | | 1. Inappropriate demands for payment |  |  |
|  |  |  | |  |  | 1. Lack of privacy |
| Type of related bias | Imperfect test error | Imperfect test error | | Imperfect test error | Imperfect test error | Imperfect test error |
| Potential impact on prevalence estimate | - Different categorization of D&A affects the comparability of summary measures of overall D&A experienced across the studies - Underestimation of total D&A if the prevalence of excluded categories of D&A was significant | | | | | |
| 1. Summary measure of D&A | Any treatment that made woman feel humiliated or disrespected | | At least one form of disrespect and abuse | At least one form of disrespect and abuse | At least one form of disrespect and abuse | At least one form of disrespect and abuse |
| Type of related bias | Imperfect test error | | Imperfect test error | Imperfect test error | Imperfect test error | Imperfect test error |
| Potential impact on prevalence estimate | - A self-report of any perceived D&A is not comparable to a score calculated by tabulating the proportion of women who reported experiencing at least one instance of a specific type of D&A | | | | | |
| - 1. Variations in operational definitions of categories | (See Table 4) | | (see Table 4) | (see Table 4) | (see Table 4) | (see Table 4) |
| Type of related bias | Imperfect test error | | Imperfect test error | Imperfect test error | Imperfect test error | Imperfect test error |
| Potential impact on prevalence estimate | - Studies with fewer operational definitions for each category of D&A may have resulted in biased estimates of prevalence because there were fewer opportunities to answer affirmatively or fewer examples to jog women’s memories - Studies with general or vague operational definitions may have resulted in biased estimates of prevalence if there was confusion about or normalization of D&A - Prevalence estimates derived from general open-ended questions are not comparable to those resulting from a list of specific questions - Variation in operational definitions of D&A results in prevalence estimates of different phenomena, which estimates are therefore incomparable - Variations in the operationalization of D&A across instruments used for data collection via different modes within one study (i.e. for interviews with women and for direct observation) make those prevalence estimates incomparable | | | | | |
| 1. **Data Collection** |  | |  |  |  |  |
| - 1. Mode | Exit interview | | Exit Interview | - Exit interview - Follow-up interviews | Self-administered questionnaire | - Exit interview - Follow-up interviews - Observation during labor and delivery |
| - 1. Timing | Once women had been discharged from the postnatal ward | | Immediately prior to discharge from the health facilities after childbirth | - Exit interview upon discharge - Follow-up interviews 5-10 weeks postpartum | 4-6 week postpartum | Immediately prior to discharge from the health facility after childbirth, approximately three to six hours post-delivery   - Follow-up interviews 4-6 weeks post delivery |
| Type of related bias | Recall-related error | | Recall-related error | Recall-related error | Recall-related error | Recall-related error |
| Potential impact on prevalence estimate | - The potential for recall-related error is introduced for data collected shortly after childbirth due to physical fatigue and other factors immediately postpartum - The potential for recall-related error is introduced for data collected weeks later if memories faded (underestimation) or if exit interview questions primed participants (possible overestimation) | | | | | |
| - 1. Setting | Within the hospital compound in a private place | | Data collection setting not reported | - Exit interview in tents outside of the health facilities - Follow-up interviews inside the participants’ homes in private without other family members present (Participants who lived outside of the study district or in hard to reach remote areas of the district were excluded from this sample) | At the immunization  clinic of the facility | - Exit interview in a separate private room near the postnatal ward - Follow-up interviews, participants recruited by mobile phone, those who consented were interviewed in their home |
| Type of related bias | Courtesy bias | | Courtesy bias | - Courtesy bias - Differential loss to follow-up | Courtesy bias | - Courtesy bias - Differential loss to follow-up |
| Potential impact on prevalence estimate | - Courtesy bias could introduce risk of under-estimation if study participants are reluctant to report D&A in proximity to the facility where it occurred or believe that the researchers are affiliated with the facility - The potential for differential loss to follow up could introduce systematic error into the prevalence estimates if women in remote areas, outside the study area, or women with mobile phones were at differential risk for D&A compared to the general study sample | | | | | |
| - 1. Data collectors | Research assistants | | Four female data collectors not involved in the women’s care | Trained interviewers | Three female  medical interns (house officers) drawn from the obstetric unit of  the hospital | Trained social scientists |
| Type of related bias | Interview-related bias | | Interview-related bias  Courtesy bias | Interview-related bias | Interview-related bias  Courtesy bias | Interview-related bias |
| Potential impact on prevalence estimate | - Interview-related bias could affect prevalence of reported D&A if interviewers gave subtle cues to participants based on their own experiences, attitudes, or opinions - The potential for courtesy bias could introduce risk of under-estimation if study participants were reluctant to report D&A to interviewers who are affiliated with the facility | | | | | |
